# The interplay between gut microbiota and gestational diabetes mellitus: mechanisms, characteristics, and intervention strategies

**DOI:** 10.3389/fmicb.2026.1789594

**Published:** 2026-07-30

**Authors:** Feng Jiang, Jing-he Zhu, Fu-Juan Liu, Xin Chen, Lu Zheng, Jian-xiu Yu, Wei Chen

**Affiliations:** 1 Department of Endocrinology, Binhai County People’s Hospital, Binhai, China; 2 Department of Endocrinology, Affiliated Dongtai Hospital of Nantong University, Dongtai, China; 3 Department of Ultrasound, Affiliated Dongtai Hospital of Nantong University, Dongtai, China; 4 Department of Medical Laboratory, Affiliated Dongtai Hospital of Nantong University, Dongtai, China; 5 Department of Clinical Laboratory, Binhai County People’s Hospital, Binhai, China

**Keywords:** gestational diabetes mellitus, gut microbiota, microbial metabolites, therapeutic strategies, vertical transmission

## Abstract

Gestational diabetes mellitus (GDM) is a common metabolic complication during pregnancy that poses significant risks to maternal and infant health and is closely associated with gut microbiota dysbiosis. This review systematically summarizes the mechanisms, characteristic alterations, and intervention strategies related to the interplay between gut microbiota and GDM. Studies have shown that women with GDM exhibit distinct structural and compositional dysbiosis of the gut microbiota, such as an altered Firmicutes/Bacteroidetes ratio, reduced abundance of beneficial bacteria, and enrichment of pathogenic bacteria. This dysbiosis is associated with altered synthesis of metabolites such as short-chain fatty acids and bile acids, which may impair insulin signaling, compromise barrier function, and activate inflammatory responses, potentially playing a role in GDM pathogenesis. Furthermore, GDM-associated gut dysbiosis can be vertically transmitted to offspring, altering neonatal gut colonization and increasing long-term risks of metabolic and neurodevelopmental disorders. Diagnostic models based on gut microbiota features show promise for early GDM prediction. Interventions targeting the gut microbiota, including lifestyle modifications, probiotics/prebiotics supplementation, and fecal microbiota transplantation, have demonstrated potential in improving glucose metabolism by modulating microbial composition. However, current research faces challenges such as high heterogeneity, unclear causal mechanisms, and difficulties in clinical translation. Future studies should integrate multi-omics approaches and prospective cohorts to elucidate the core mechanisms of gut microbiota in GDM and advance personalized prevention and management strategies.

## 1 Introduction

Gestational diabetes mellitus (GDM) is defined as glucose intolerance that is first diagnosed or recognized during pregnancy and represents a significant metabolic complication of gestation (Diabetes Care, 2022). GDM poses a direct threat to maternal health, increasing the risk of adverse pregnancy outcomes such as gestational hypertension, polyhydramnios, preterm birth, and dystocia. Furthermore, GDM exerts profound long-term effects on the health of the fetus and offspring. In the short term, it can lead to fetal growth restriction, macrosomia, neonatal hypoglycemia, and respiratory distress syndrome. In the long term, it significantly elevates the offspring’s future risk of obesity, type 2 diabetes, metabolic syndrome, and neurodevelopmental disorders, thereby causing persistent disruption to the health trajectories of both the mother and child across two generations (Kunysz et al., 2021; Słupecka-Ziemilska et al., 2020).

According to the diagnostic criteria established by the International Association of Diabetes in Pregnancy Study Group, approximately 21 million newborns worldwide are affected by hyperglycemia in pregnancy among women of reproductive age (20–40 years) annually, with GDM accounting for up to 80.3% of these cases. The overall global prevalence of GDM has reached 14% (Chinese Endocrinologist Association, 2021; Wang et al., 2021). Furthermore, a meta-analysis focusing on mainland China reported a GDM prevalence of 14.8%, which is slightly higher than the global average (Sweeting et al., 2024). Against the backdrop of rising global obesity rates, the incidence of GDM and the associated rates of pregnancy-related and perinatal complications have also increased, imposing a significant burden on maternal and child healthcare systems and public health resources (Zhou et al., 2022).

In recent years, with the advancement of microbiome and metabolomics research, the gut microbiota, often regarded as a hidden organ that regulates host nutrient absorption, metabolic balance, and immune activation, has become a focus in studies investigating its association with GDM (Flores Ventura et al., 2025). Research has confirmed that women with GDM commonly exhibit dysbiosis of the gut microbiota, characterized by an imbalance in microbial composition, reduced abundance of beneficial bacteria, and enrichment of pathogenic bacteria. This dysbiosis can further disrupt host insulin signaling and exacerbate inflammatory responses by affecting the synthesis of metabolites such as short-chain fatty acids (SCFAs), bile acids, and branched-chain amino acids (BCAAs), thereby contributing to the onset and progression of GDM. Furthermore, GDM can influence the vertical microbial transmission from mother to infant, altering the colonization and developmental trajectory of the offspring’s gut microbiota and contributing to the intergenerational transmission of health risks. This review will systematically summarize the association between GDM and gut microbiota characteristics, the metabolic profiles associated with GDM, and intervention strategies targeting the gut microbiota. It will also analyze current research challenges and future directions, aiming to provide theoretical foundations and clinical references for elucidating the mechanisms, early screening, and precise intervention of GDM.

This is a narrative review. A literature search was performed in PubMed, Web of Science, and CNKI for articles published between January 2014 and April 2026. The search strategy combined three groups of terms using Boolean operators (OR within groups, AND across groups). The condition terms included “gestational diabetes mellitus” OR “GDM”; the mechanism/target terms included “gut microbiota” OR “intestinal microbiome” OR “gut microbiome” OR “dysbiosis” OR “metabolomics” OR “metabolism” OR “short chain fatty acids” OR “SCFAs” OR “bile acids”; and the intervention terms included “treatment” OR “fecal microbiota transplantation” OR “FMT” OR “probiotics” OR “prebiotics” OR “synbiotics” OR “diet” OR “lifestyle modification” OR “exercise.” The final search query was (condition terms) AND (mechanism/target terms OR intervention terms). Inclusion criteria were: (1) studies involving human GDM patients or GDM animal models; (2) original research (cross-sectional, cohort, randomized controlled trials, or animal experiments), systematic reviews, or meta-analyses; and (3) studies explicitly addressing gut microbiota or metabolite alterations, mechanisms, diagnostic value, or intervention effects. Exclusion criteria were non-English or non-Chinese articles, case reports, conference abstracts, and articles lacking core data. A total of 96 articles met the inclusion criteria and formed the basis of this review.

## 2 Gut microbiota characteristics and alterations associated with GDM

### 2.1 Gut microbiota dysbiosis in GDM and its characteristics as potential biomarkers

Individuals with GDM exhibit specific alterations in the structure and function of their gut microbiota; these alterations also serve as potential biomarkers for the condition. However, existing findings are highly heterogeneous, characterized by dynamic changes during pregnancy and significant population specificity, which may be attributed to variations in population demographics (dietary patterns, medication, etc.), geographical locations, sample size, sample collection time during pregnancy and methodological approaches (Table 1).

**Table 1:** Alterations in gut microbiota in GDM.

Studies	Area	Subjects	Alterations in gut microbiota	Research method
Cui et al. (2024)	China	GDM (*n* = 27)	Increased: At the phylum levels: Firmicutes, Actinobacteria At the genus level: *Bifidobacterium pseudocatenulatum, Bifidobacterium adolescentis, Bifidobacterium longum* Decreased: At the phylum levels: Bacteroidetes	Metagenomic sequencing
Valencia-Castillo et al. (2024)	Colombia	GDM (*n* = 5)	Increased: At the phylum levels: Firmicutes At the genus level: unclassified *Paenibacillaceae, Paenibacillus, Staphylococcus, Brevibacillus, Bacillus* Decreased: At the phylum levels: Bacteroidetes, Proteobacteria At the genus level: *Alistipes*, *Phascolartobacterium*, *Bacteroides*, *Coprobacillus*, *Allobaculum*, *Megamonas*, *Bilophila*, *Parabacteroides*	16S rRNA
Xiao et al. (2024)	China	GDM (*n* = 68)	Increased: At the genus level: *Escherichia*, *Klebsiella* Decreased: At the genus level: *Bacteroides*, *Bifidobacterium*, *Coprococcus*, *Ruminococcus*	16S rRNA
Liang et al. (2022)	China	GDM (*n* = 35)	Increased: At the phylum levels: *Bacteroidetes*, *Proteobacteria*, *Fusobacteria* At the family level: *Bacteroidaceae*, *Prevotellaceae*, *Acidaminococcaceae*, *Veillonellaceae*, *Lachnospiraceae*, *Ruminococcaceae*, *Enterobacteriaceae*, *Tannerellaceae* At the genus level: *Bacteroides*, *Prevotella_9*, *Megamonas*, *Phascolarctobacterium*, *Lachnospiraceae*, *Megasphaera*, *Prevotella_2*, *Parabacteroides, Bacteroides, Lachnoclostridium* Decreased: At the phylum levels: *Firmicutes*, *Verrucomicrobia*, *Synergistetes*, *Tenericutes*	16S rRNA
Dualib Fernandes et al. (2022)	Brazil	GDM (*n* = 56)	Increased: At the genus level: *Bacteroides*	16S rRNA
Vavreckova et al. (2022)	Czech Republic	GDM (*n* = 82)	Increased: At the classes levels: Negativicutes, Clostridia At the family level: *Oscillospiraceae*, *Enterococcus*, *Erysipelotrichaceae UCG-003, Bilophila*, *Leuconostoc*, *Streptococcus* Decreased: At the classes levels: D*esulfovibrionea*, *Bacilli*	16S rRNA
Sun et al. (2023)	China	GDM (*n* = 120)	Increased: At the genus level: *Bacteroides massiliensis, E. ramulus, Anaerostipes hadrus, B. massiliensis* Decreased: At the genus level: *Ruminococcus bromii, Alistipes putredinis*, *Bacteroides ovatus, R. bromii*, *A. putredinis*, *Bi. dentium*	Shotgun metagenomic profiling
Hu L. et al. (2025)	China	GDM (*n* = 10)	Increased: At the genus level: *Fusicatenibacter*, *CAG-352*, *Moryella*, and *norank_f__Lachnospiraceae*	16S rRNA
Wang J. et al. (2024)	China	GDM (*n* = 90)	Increased: At the genus level: *Parabacteroides distasonis* Decreased: At the genus level: *Akkermansia muciniphila*, *Coprococcus eutactus*, *Enterorhabdus caecimuris, Dialister* sp. *CAG 357, Adlercreutzia equolifacien*, *Asaccharobacter celatus*	Metagenomic sequencing
Liu Y. et al. (2023)	China	GDM (*n* = 49)	Increased: At the phylum levels: *Firmicutes* At the genus level: *unidentified_Lachnospiraceae*, *Blautia*, *Roseburia, Parabacteroides, Lachnospira, Megamonas* Decreased: At the phylum levels: *Actinobacteria* At the genus level: *Bifidobacterium*	16S rRNA
Gupta et al. (2024)	Singapore	GDM (*n* = 53)	Increased: At the phylum levels: *Actinobacteriota*, *Firmicutes*, *Fusobacteriota* At the genus level: *Collinsella, Blautia, Ruminococcus, Ruminococcus gnavus, Collinsella, Blautia, Bifidobacterium, Dorea, Roseburia, Coprococcus, Anaerostipes, Ruminococcus gnavus group, Ruminococcus torques group, Eubacterium hallii group, Romboutsia, Fusicatenibacter, Clostridium* sensu stricto *1, Agathobacter, Ruminococcus, Megasphaeraruminococcus torques, Eubacterium hallii groups* Decreased: At the phylum levels: *Bacteroidota* At the genus level: *Akkermensia, Bacteroides, Acidaminococcus, Escherichia-Shigella, Klebsiella, Lachnospiraceae NK4A136*	16S rRNA

At the phylum level, an imbalance in the Firmicutes/Bacteroidetes (F/B) ratio is a core feature, though its direction varies across studies. Whole-genome shotgun sequencing of 27 mid-pregnancy GDM patients in Beijing revealed a significantly higher F/B ratio compared with the normal glucose tolerance (NGT) group (Cui et al., 2024). Similarly, a Colombian study of five GDM pregnant women reported a Firmicutes-dominated microbiota with reduced alpha diversity and markedly decreased relative abundances of Bacteroidetes and Proteobacteria (Valencia-Castillo et al., 2024). Conversely, a study from Xiamen, China, found significant enrichment of Proteobacteria, *Escherichia*, and *Klebsiella*, alongside reduced abundances of Firmicutes, Bacteroides, and *Bifidobacterium* (Xiao et al., 2024). Studies from Guangdong, China, and Brazil observed an increased abundance of Bacteroides in GDM groups, although overall structural differences did not reach statistical significance (Dualib, Taddei et al., 2022; Liang et al., 2022). A study of 300 healthy Iranian women in early pregnancy further indicated that higher abundances of Bacteroidetes and Actinobacteria were associated with a reduced risk of developing GDM in mid-pregnancy, while an increased F/B ratio correlated with a higher risk of glucose intolerance (Mousavi et al., 2025).

At the genus level, some consistent alterations have been identified. The abundances of genera such as *Blautia*, *Enterococcus*, and *Erysipelotrichaceae*_UCG-003 are commonly elevated in GDM groups, whereas *Bifidobacterium* and certain *Ruminococcus*-related taxa (e.g., *Ruminococcus bromii*, *Ruminococcaceae*_UCG-002, *Ruminococcaceae*_UCG-005) generally show a decreasing trend (Liang et al., 2022; Sun et al., 2023; Vavreckova et al., 2022). However, these patterns are not universally observed. Wei et al. (2021) reported increased rather than decreased abundances of *Ruminococcus bromii*, along with elevated *Clostridium colinum* and *Streptococcus infantis*, the latter positively correlating with glucose levels. Furthermore, a study by Hu L. et al. (2025) found no significant difference in alpha or beta diversity between GDM and healthy pregnant women, with compositional differences evident only at the genus level. The gut microbiota of healthy pregnant women was dominated by *Faecalibacterium*, *Butyricicoccus*, and *Lachnospiraceae*_UCG-004, whereas GDM patients exhibited the highest relative abundance of *Blautia*, along with increased abundances of *Fusicatenibacter*, CAG-352, *Moryella*, and *norank_f_Lachnospiraceae*. Geographic differences further contribute to this heterogeneity. In non-obese Japanese GDM women, enrichment of *Romboutsia* was linked to insulin resistance, reduction of *Collinsella* might reflect impaired insulin secretion, and an increase in *Akkermansia* could be associated with dietary intervention response (Tanaka et al., 2021).

Longitudinal studies have elucidated the temporal dynamics of GDM-associated gut microbiota. In the first trimester, a reduction in microbial diversity is already evident, with decreased levels of *Akkermansia muciniphila* and increased pathogenic bacteria such as *Escherichia coli* (Wang S. et al., 2024). A prospective cohort study in Chinese women demonstrated that pregnancy-induced alterations in the maternal gut microbiota were primarily driven by GDM; *Bacteroides vulgatus* and *Ruminococcus gnavus* were significantly enriched in the first trimester and positively correlated with insulin signaling and lipopolysaccharide (LPS) biosynthesis pathways (Li M. et al., 2023). Women with recurrent GDM exhibited a more staged dysbiosis. Their first trimester was characterized by enrichment of pro-inflammatory genera like *Streptococcus* and *Enterobacter* and depletion of beneficial ones like *Bacteroidetes*. By the second trimester, they showed reductions in protective taxa such as *Lactobacillaceae* and *Desulfovibrio*, alongside a significant decrease in microbial network tightness, indicating impaired ecological interactions (Zheng et al., 2025). During the mid-to-late stages of pregnancy, compositional differences become more pronounced; GDM mothers showed higher abundances of Firmicutes and unidentified *Lachnospiraceae*, and lower abundance of Actinobacteria (Liu N. et al., 2023). A longitudinal analysis by Wu X. et al. (2025) found that although overall gut microbiota diversity did not significantly differ between GDM and healthy pregnancies, the relative abundances of *Ruminococcaceae*_UCG-002 and *Odoribacter* showed specific alterations throughout gestation in the GDM group. Mendelian randomization analysis from the same study confirmed *Prevotella*_9 as a risk factor for GDM and *Methanobrevibacter* as a protective factor, providing potential targets for microbiota-based interventions. Notably, a multi-ethnic study of Chinese, Malay, and Indian women in Singapore revealed shared gut microbial features among GDM women across these ethnicities, with genera such as *Collinsella*, *Blautia*, and *Ruminococcus* consistently enriched, and this dysbiosis persisted despite dietary and lifestyle interventions (Gupta et al., 2024) (Tables 1, 2).

**Table 2:** Alterations in infant gut microbiota associated with maternal GDM.

Studies	Area	Subjects	Alterations in infant gut microbiota	Research method
Valencia-Castillo et al. (2024)	Colombia	GDM mother–Infant (*n* = 5)	Increased: At the phylum levels: Firmicutes, Decreased: At the phylum levels: Bacteroidetes At the genus level: *Actinobacteria,* *Chloroflexi, Synergistetes, Verrucomicrobia, Bifidobacterium*, *Sutterella, Serratia*, *Flavonifractor* sp., *Enterobacteriaceae*, *Lactobacillus* sp.	16S rRNA
Low et al. (2025)	Singapore	GDM mother– Infant (*n* = 16)	Increased: At the phylum levels: *Firmicutes*, *Actinobacteriota* At the genus level: *Bifidobacterium*, *Blautia*, *Collinsella*, *Clostridium sensu stricto* 1, *Acinetobacter*, *Bacteroides*, *Pseudomonas*, *Staphylococcus*, *Enterococcus*, *Eubacterium halli group*, *Erysipelotrichaceae* UCG-003, *Sellimonas, Bifidobacterium*, *Bacteroides*, *Blautia*, *Intestinibacter*, *Rothia*, *Clostridium sensu stricto* 1, *Staphylococcus*, *Eubacterium hallii group*, *Streptococcus*, *Ruminococcus torques group* Decreased: At the phylum levels: *Proteobacteria,* *Bacteroidota*	16S rRNA
Liu J. et al. (2024)	China	GDM mother– Neonate (*n* = 44)	Increased: At the phylum levels: *Actinobacteria* At the genus level: *Phyllobacterium,* *Rhizobiaceae, Rhizobiales, Bifidobacterium, Blautia, Collinsella, Clostridium sensustricto 1* Decreased: At the phylum levels: *Bacteroidota* *Proteobacteria, Oxophotobacteria* At the genus level: *Escherichia-Shigella,* *Bacteroides, Sutterella, Alistipes*	16S rRNA
Qin et al. (2022)	China	pregnant mice (*n* = 7) Pups (*n* = 13)	Increased: At the genus level: *Anaeroplasma*, *Eubacterium_brachy* group, *Eubacterium_xylanophilum* group, *Lachnospiraceae* UCG 006, *Ruminiclostridium 5*, *Ruminococcaceae* UCG_003, *Oscillibacter*, *Lachnoclostridium*, *Ruminococcaceae* UCG_009, *Turicibacter*, *Bacteroides*, *Ruminococcaceae* UCG_013, *Enterorhabdus*, *Lachnospiraceae* NK4A136 group, *Globicatella*, *Enterococcus*, *Desulfovibrio*, *Anaerovorax*, DNF00809, *Harryflintia*, *Romboutsia* Decreased: At the genus level: *Akkermansia, Parvibacter,* *Ruminococcus torques, Catenibacterium,* *Candidatus Stoquefichus*	16S rRNA
Zhang, Tan et al., 2025	China	neonates (*n* = 16)	Increased: At the phylum levels: Bacteroidetes At the genus level: *Bacteroides*, *CAG_352*, *Escherichia-Shigella*, NK4A214_group, *Peptostreptococcaceae*, *Flavobacteriales*, *Lachnospiraceae_UCG_010* Decreased: At the phylum levels: Actinobacteria, Proteobacteria At the genus level: *Pelomonas*, *Enterococcus*, *Streptococcus*, *Prevotella*	16S rRNA
Mo et al. (2025)	China	Infants (*n* = 35)	Increased: At the family levels: *Coriobacteriaceae*, *Clostridiaceae*, *Erysipelotrichaceae*, *Erysipelatoclostridiaceae* At the genus level: *Collinsella, Clostridium_sensu_stricto_1, Erysipelatoclostridium* Decreased: At the family levels: *Lactobacillaceae* At the genus level: *Pediococcus*	16S rRNA
Hu Y. et al. (2025)	China	Neonates (*n* = 15)	Increased: At the phylum levels: Proteobacteria At the genus level: *Stenotrophomonas*, *Chryseobacterium*, *Pseudescherichia* Decreased: At the phylum levels: Actinobacteria, Firmicutes At the genus level: *Bifidobacterium*, *Blautia*, *Fusicatenibacter, Collinsella*, *Anaerostipes, Faecalibacterium*, *Pelomonas*, *Roseburia*, *Mediterraneibacter*, *Agathobacter*	16S rRNA
Su et al. (2025)	China	Neonates (*n* = 29)	Decreased: At the phylum levels: Firmicutes At the genus level: *Eubacterium*, *Eubacterium hallii*	16S rRNA
Xiao et al. (2024)	China	Neonates (*n* = 68)	Increased: At the phylum levels: *Proteobacteria* At the genus level: *Escherichia*, *Klebsiella* Decreased: At the phylum levels: *Firmicutes* At the genus level: *Bacteroides*, *Bifidobacterium*, *Coprococcus*, *Ruminococcus, Clostridium, Coriobacteriaceae, Collinsella*	16S rRNA
Zhu et al. (2022)	China	Neonates (*n* = 60)	Increased: At the genus level: *Xanthobacter*, *Cytophaga*, *Serratia*, *Actinomyces* Decreased: At the phylum levels: *Proteobacteria* At the genus level: *Enhydrobacter*, *Psychrobacter*, *Aerococcus*, *Faecalibacterium*, *Herbaspirillum*, *Pelomonas*, *Burkholderia-Caballeronia-Paraburkholderia*	16S rRNA
Nieto-Ruiz et al. (2023)	Spain	18 months children (*n* = 48)	Increased: At the genus level: *Fusicatenibacter,* *Butyricococcus, Barnesiella, Megasphaera* Decreased: At the phylum levels: *Firmicutes* At the genus level: *unclass_Clostridiales, Flavonifractor*	16S rRNA
Valdez-Palomares et al. (2024)	Mexico	Infant (*n* = 14)	Increased: At the genus level: *Bacteroides* Decreased: At the family level: **Enterobacteriaceae** At the genus level: *Veillonella, Clostridiales*	16S rRNA
Zhang, Wang et al. (2025)	China	Neonates (*n* = 16)	Increased: At the phylum levels: Bacteroidetes At the genus level: *Bacteroides*, *Escherichia-Shigella* Decreased: At the phylum levels: Actinobacteria, Proteobacteria At the genus level: *Pelomonas*, *Enterococcus*, *Streptococcus*, *Prevotella, NK*4A214_group, *Peptostreptococcaceae*, *Flavobacteriales*, *CAG_352*, *Lachnospiraceae_UCG_010*	16S rRNA
Liu et al. (2025)	China	Neonates (*n* = 114)	Increased: At the phylum levels: *Firmicutes* At the genus level: ***Ruminococcus, Eubacterium, Butyricicoccus,** Pseudoxanthomonas, Roseomonas, Hymenobacter* Decreased: At the phylum levels: *Bacteroidetes* At the genus levels: *Eubacterium*, *Eubacterium hallii, Escherichia-Shigella, Klebsiella, Prevotella, Mitsuokella*	16S rRNA
Petitclerc et al. (2025)	Canada	Infants (*n* = 17)	Increased: At the phylum levels: Firmicutes At the family levels: *Veillonellaceae*	16S rRNA
Song et al. (2023)	China	Infants (*n* = 34)	Increased: At the phylum level: Actinobacteria At the class levels: *Coriobacteriia, Actinomycetia,* At the order levels*: Bifidobacteriales*, *Coriobacteriales* At the family levels: *Bifidobacteriaceae* *Coriobacteriaceae* At the genus level: *Bifidobacterium,* *Clostridium* Decreased: At the phylum levels: *Proteobacteria* At the class levels: *Gammaproteobacteria* At the order levels: *Enterobacterales* At the family levels: *Enterobacteriaceae,* Oxalobacteraceae At the genus level: *Ralstonia*	16S rRNA
Chieu et al. (2024)	Canada	12 months Infants (*n* = 16)	Increased: At the phylum level: *Fusobacterium* Decreased: At the family levels: *Streptococcus, Bacteroidaceae*	16S rRNA
Sato et al. (2014)	China	Neonates (*n* = 10) vaginal delivery	*Increased:* *At the genus level: Bacteroides xylanisolvens, Bacteroides thetaiotaomicron, Phocaeicola vulgatus, Escherichia coli_D, Clostridium_AQ innocuum*	16S rRNA
		Neonates (*n* = 24) cesarean section	Increased: At the genus level: *Veillonella parvula_A*, *V. infantium*, *V. dispar*, *Staphylococcus hominis*, *Clostridium_P perfringens*	
		Neonates (*n* = 10) breast feeding	At the genus level: *Corynebacterium kefrresidentii*, *Staphylococcus, lugdunensis*, *Streptococcus infantis_I*, *Rothia mucilaginosa_B*	
		Neonates (*n* = 24) mixed feeding	At the genus level: *Klebsiella pneumoniae*	
Dualib, Taddei et al. (2022)	Brazil	2–6 months Infants (*n* = 45) breast feeding	Increased: At the genus level: *Bacteroides,* *Staphylococcus*	16S rRNA

The composition of GDM-associated gut microbiota is further modified by host factors, underscoring its population specificity. BMI is a key modifier: a nested case–control study in China reported decreased *Bacteroides_H* and *Acetatifactor* in the first trimester and enrichment of inflammatory genera such as *Fusobacteriota* in the second trimester among GDM women; overweight/obese GDM patients exhibited even higher levels of pro-inflammatory bacteria like *Streptococcus* and upregulated inflammatory pathways (Zhong et al., 2024). In a large prospective study of 1,820 participants, Mei et al. (2025) observed significantly altered abundances of *Blautia*, *Anaerostipes*, *Synergistes*, and *Christensenellaceae_R_7_group* in obese GDM women compared to non-obese GDM women, and seven genera including *Odoribacter* were associated with both oral glucose tolerance test (OGTT) glucose levels and pre-pregnancy BMI. Importantly, gut microbiota alterations can precede clinical symptoms. Before 20 weeks of gestation, both GDM and gestational hypertensive disorders of pregnancy (HDP) patients already exhibited distinct dysbiosis patterns, with shared enrichment of *Synergistaceae*. Specifically, the GDM group had increased *Bacteroidetes*, while the HDP group had increased *Actinobacteria* and decreased *Bacteroidetes*, suggesting that microbiota biomarkers may have cross-disease relevance for metabolic disorders (Zuo et al., 2025).

In summary, GDM is associated with gut microbiota dysbiosis characterized by alterations in microbial structure, diversity, and specific bacterial taxa, which also show promise as early biomarkers. However, significant heterogeneity exists across studies, reflecting differences in dietary patterns, sequencing platforms, gestational age at sampling, population characteristics, sample size, and the extent of control for pre-pregnancy BMI and antibiotic exposure. These methodological differences limit the interpretability of inconsistent findings and must be considered when comparing results. Future rigorous, multi-center prospective studies are required to validate robust biomarker panels and facilitate clinical translation.

### 2.2 Construction and performance of GDM diagnosis and prediction models

Based on these characteristic microbiota biomarkers, and integrating multi-dimensional data such as clinical indicators, metabolites, and inflammatory factors, researchers have developed various diagnostic and predictive models for GDM (Table 2). Their performance has been validated in different cohorts. In terms of model optimization, Popova et al. (2025) constructed a predictive model integrating continuous glucose monitoring, dietary records, clinical indicators, and gut microbiota data. They demonstrated that incorporating microbiota features increased the model’s explained variance for postprandial glucose peaks in GDM patients from 34 to 42%, confirming the adjunctive value of microbiota in optimizing glucose management. For risk prediction, Guan et al. (2025) developed a random forest model based on 10 bacterial species and 10 metabolites to predict pre-pregnancy GDM risk in 193 infertile women undergoing frozen embryo transfer. The model achieved an area under the receiver operating characteristic curve (AUC) of 0.712. Zhang et al. (2024) employed a random forest algorithm to screen 26 key biomarkers, including 5 microbial species, 1 contig, 15 genes, and 5 single-nucleotide variants. Their model attained a cohort AUC of 0.860 for diagnosing GDM, with a validation cohort AUC of 0.730, significantly outperforming models using single-type biomarkers. Multi-cohort studies further confirm the model robustness. Pinto et al. found that first-trimester changes precede GDM diagnosis by several months, characterized by gut dysbiosis (e.g., reduced Prevotella), elevated pro-inflammatory cytokines (IL-6, IL-8), and metabolic disturbances. FMT experiments confirmed that this dysbiotic microbiota could induce impaired glucose tolerance in mice. Based on this, a machine learning model integrating first-trimester microbial markers, inflammatory factors, and clinical data achieved an AUC of 0.83 for predicting GDM (Pinto et al., 2023). Other studies have sought higher predictive performance by optimizing biomarker combinations. Research by Liang et al. showed that combining 20 specific bacterial genera with GLU levels yielded a predictive AUC of 0.94 for GDM, outperforming models using only microbiota features or those combined with GLP-1, insulin, or HbA1c (Liang et al., 2022). A diagnostic model based on genus-level biomarkers such as *Porphyromonas*, *Fenollaria*, *Klebsiella*, *Prevotella*, and *Corynebacterium* achieved an even higher AUC of 0.982, demonstrating high sensitivity and specificity for early GDM prediction (Yao W. et al., 2025). Utilizing gut microbiota features shows significant potential for the early warning and auxiliary diagnosis of GDM, especially when integrated with multi-omics and machine learning methods. However, current research faces core challenges, including inconsistent biomarkers, limited model generalizability, and difficulties in clinical translation. Future efforts require large-scale, standardized multi-center studies to identify robust biomarker panels and advance their translation into clinical tools.

Despite promising performance, microbiota-based models face several translational barriers, including limited external validation, uncertain reproducibility across populations and sequencing platforms, and the need for integration with conventional clinical markers. The feasibility of first-trimester risk prediction also remains to be established. Addressing these challenges is essential before microbiome-based diagnostic tools can be translated into routine clinical practice.

### 2.3 Potential biological mechanisms of gut microbiota in influencing GDM

**Figure 1:**
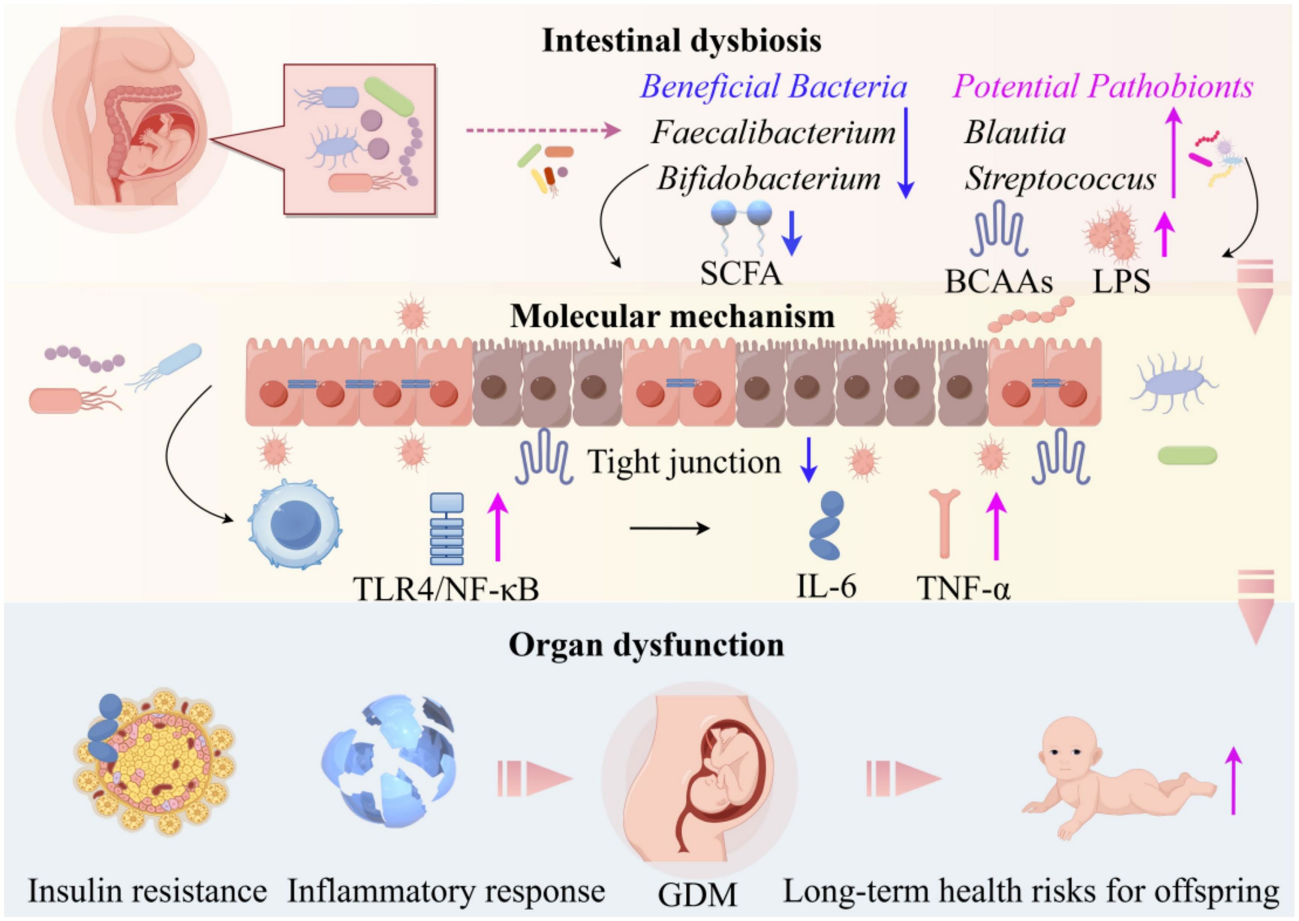
Mechanisms of the microbiota in the development of GDM. (By Figdraw).

The gut microbiota has been implicated in the pathogenesis and progression of GDM through multiple interconnected pathways, primarily by regulating metabolic pathways, compromising barrier function, activating inflammatory and immune responses, and disrupting the stability of mother-offspring microbial colonization. Concurrently, factors such as microbial genetic variations and bacteriophages may also play indirect roles. These mechanisms are interlinked and collectively mediate the disease process (Figure 1).

#### 2.3.1 Metabolic dysregulation and insulin resistance

Aberrations in gut microbiota structure may influence insulin sensitivity through changes in multiple glucose and lipid metabolic pathways, and have been proposed as a potential mechanism in GDM pathogenesis. *Bifidobacteriaceae* may contribute to improved glucose metabolism through the regulation of quorum sensing pathways and the synthesis pathways of surface compounds in Gram-negative bacteria (such as COLANSYN-PWY, PWY-7323), and has been proposed as a key regulatory factor after GDM intervention (Cui et al., 2024). Conversely, GDM-associated dysbiosis (e.g., enrichment of *Collinsella* and *Blautia*) may activate carbohydrate metabolism pathways like the pentose phosphate pathway, a pattern that has been associated with insulin resistance. Notably, this dysbiosis exhibits stability across different ethnicities (Gupta et al., 2024). The regulation of metabolic pathways by the gut microbiota exhibits dynamic changes across different stages of pregnancy. In GDM, alterations in gut microbiota have been associated with a dynamic shift in butyrate synthesis pathways, characterized by activation in early pregnancy followed by suppression later on. Concurrently, the mevalonate pathway becomes overactive in late pregnancy. These collective changes result in a serum butyrate level that initially rises and then falls, while concentrations of mevalonate, estradiol, and progesterone are significantly elevated. This imbalance has been proposed to contribute to insulin resistance by interfering with insulin signaling and exacerbating inflammatory responses (Lyu et al., 2023). Furthermore, increased abundance of the genus *Clostridium* in GDM patients is significantly associated with dysregulated phenylalanine metabolism, accompanied by elevated levels of metabolites such as phenylalanylglycine and uracil (Lin et al., 2024). Conversely, reduced abundance of beneficial bacteria like *Faecalibacterium* has been associated with lower SCFA biosynthetic capacity and reduced serum dopamine levels and related pathway activity, potentially contributing to impaired dopaminergic signaling. These metabolic abnormalities may collectively promote the pathological progression of GDM (Ye et al., 2023).

#### 2.3.2 Impaired barrier function and abnormal exposure to microbial components

It is noteworthy that the metabolic dysregulation described above, particularly alterations in key metabolites like SCFAs, can directly or indirectly compromise intestinal barrier integrity and act as pro-inflammatory signals that initiate immune responses, suggesting a potential link between metabolic disturbances and subsequent barrier dysfunction and inflammatory states. Indeed, GDM is associated with impaired barrier function at multiple levels, including the maternal intestinal barrier, the placental barrier, and the offspring’s intestinal barrier. In GDM mothers, increased intestinal permeability has been documented; for instance, the antibody response to *Bifidobacterium adolescentis* correlates negatively with intestinal permeability markers, providing direct evidence of maternal gut barrier dysfunction (Vorobjova et al., 2025). This compromised maternal gut barrier facilitates the translocation of microbial components such as LPS into the systemic circulation. Significantly elevated LPS levels have been confirmed in both maternal and umbilical cord blood of GDM pregnancies, indicating that microbial components can reach not only the maternal circulation but also the fetal compartment (Liu J. et al., 2024; Sato et al., 2014). Such translocation represents a fundamental prerequisite for the subsequent exposure of the placenta and fetus to microbial products. Regarding the placental barrier, reduced occludin expression has been reported in placentas from GDM pregnancies, which may further facilitate the passage of microbial components from maternal blood into the fetal environment (Liu J. et al., 2024). Concurrently, levels of innate immune markers such as Toll like receptors 4 (TLR4), TLR5, IL-22, and IL-23 are elevated in the placentas of both GDM patients and GDM mouse models, indicating activation of the innate immune system (Liu J. et al., 2024). GDM not only affects the maternal intestinal and placental barriers but may also indirectly impair offspring intestinal barrier function by altering the composition and stability of their gut microbiota. Offspring of GDM mothers exhibit higher levels of intestinal fatty acid binding protein (a marker of intestinal barrier damage) and anti-*β*-lactoglobulin IgA antibodies. Moreover, while children inherently have higher intestinal permeability than adults, only the offspring of GDM mothers show a specific increase in anti-β-lactoglobulin IgA (Vorobjova et al., 2025). This altered barrier function is also partly reflected in reduced stability of gut microbiota colonization. Offspring exposed to GDM exhibit poorer colonization capacity for beneficial bacteria (e.g., Lactobacillus) while showing more stable colonization by potential pathogens (e.g., *Escherichia coli*) (Liu J. et al., 2024). The weakening of barrier function at multiple levels may facilitate the abnormal translocation of gut microbiota and metabolites like LPS. This not only increases exposure to microbial products but also serves as a key link in triggering and amplifying systemic inflammation and immune abnormalities.

#### 2.3.3 Systemic inflammation and abnormal immune activation

Gut microbiota dysbiosis has been associated with exacerbated insulin resistance and may promote the development of GDM by activating inflammatory and immune pathways. On one hand, the enrichment of GDM-associated pro-inflammatory bacteria (e.g., *Alistipes shahii*) has been associated with excessive activation of metabolic inflammation. Conversely, a decreased abundance of beneficial bacteria (e.g., *Faecalibacterium*) has been linked to reduced anti-inflammatory capacity, suggesting a state of inflammatory imbalance (Ye et al., 2023). On the other hand, elevated levels of LPS, a key pro-inflammatory mediator, in both GDM mothers and fetuses, can further activate innate immune receptors such as TLR4, amplifying the inflammatory response (Liu J. et al., 2024). In addition, GDM may also affect infants’ immunity and development in a gender-specific manner, specifically manifesting as associations between abnormal head circumference in male infants and dysbiosis of bacteria such as *Clostridium paraputrificum* (Wang S. et al., 2024). The gut microbiota plays a crucial role in neonatal immune system development. Genera such as *Bacteroides* and *Phocaeicola*, which are abundant in vaginally delivered newborns, aid in immune system maturation. Microbial alterations associated with cesarean delivery and GDM may disrupt normal immune development, increasing the risk of allergies and infections (Shi et al., 2024). This persistent state of inflammation and immune activation worsens insulin resistance, creating a vicious cycle with the aforementioned metabolic dysregulation. Simultaneously, the inflammatory environment can further alter the gut microenvironment, affecting microbial colonization and function, thereby forming a complex feedback network.

#### 2.3.4 Indirect effects of microbial genetic variations and other factors

In addition to directly regulating metabolism and immunity, the genetic characteristics of gut microbiota and other factors such as bacteriophages may also contribute to GDM pathogenesis. The gut microbiota of GDM patients exhibits a significant increase in single-nucleotide variants and insertions/deletions, particularly C > T and T > C base substitutions. These genetic variations have been associated with disruptions in sphingolipid and galactose metabolism pathways and have been linked to impaired gene function and altered SCFA profiles (Zhang et al., 2024). Beyond microbial genetic variations, bacteriophages represent another factor that may indirectly influence GDM. Bacteriophage diversity positively correlates with bacterial diversity, and maternal GDM status has been shown to alter the diversity and structure of infant gut bacteriophages, thereby indirectly shaping the neonatal gut microbiota (Shi et al., 2024). This abnormal microbial shaping may further exacerbate offspring metabolic and immune risks. Together, microbial genetic variations and phage-mediated remodeling establish initial conditions that interact with the core mechanisms of metabolism, barrier function, and inflammation, forming a multi-layered and interconnected pathogenic network.

The gut microbiota is deeply involved in the onset and progression of GDM through multiple pathways, including metabolic regulation, barrier function, inflammatory immunity, and genetic variations. These mechanisms do not operate in isolation but constitute a dynamic, interconnected network. Future research should further integrate multi-omics approaches and longitudinal designs to systematically elucidate the interactions within this network, thereby providing a theoretical foundation for precise, microbiota-based prevention and management of GDM.

### 2.4 Vertical transmission of gut microbiota and its impact on offspring

Maternal GDM not only affects the mother’s own gut microbiota but may also disrupt the early colonization and development of the offspring’s gut microbiota through mechanisms such as vertical transmission. This disturbance poses potential risks to both the short-term and long-term health of the offspring.

#### 2.4.1 Evidence for vertical transmission

Early evidence demonstrated GDM-associated dysbiosis in both maternal and neonatal microbiota, suggesting shared microbial features potentially driven by vertical transmission (Wang et al., 2018). A significant correlation exists between the gut microbiota of mothers with GDM and their offspring, providing further direct support for vertical transmission. Research has found that the abundances of beneficial bacteria, such as *Bifidobacterium* and *Sutterella*, are significantly reduced in both GDM mothers and their infants. This suggests that GDM may induce infant gut dysbiosis through microbial vertical transmission (Valencia-Castillo et al., 2024). The study by Low et al. (2025) further confirmed that signature bacterial genera from GDM mothers (e.g., *Blautia*, *Collinsella*, *Bifidobacterium*, *Romboutsia*, and *Clostridium sensu stricto 1*) could be consistently detected in the meconium and 6-week-old stool samples of their infants. Source tracking analysis indicated that the maternal contribution to the infant’s gut microbiota was higher at 6 weeks than in the meconium, providing supportive evidence for vertical transmission. Furthermore, Liu J. et al. (2024) observed synchronized changes in the abundance of 14 bacterial genera in paired mother-infant samples from GDM pregnancies. Genera such as *Phyllobacterium*, *Qipengyuania*, and *Faecalitalea* were significantly increased in both mothers and infants, while *Roseburia*, *Paraprevotella*, and *Rhodococcus* were decreased. Notably, an inverse trend in gut microbiota alpha diversity was observed between GDM mothers and their infants. GDM mothers typically exhibited a higher Shannon index than healthy mothers, whereas their infants showed a lower Shannon index compared to infants of healthy mothers. This finding provides a new perspective for investigating the mechanisms by which GDM disrupts intergenerational microbiota transmission (Liu J. et al., 2024). However, other studies have reported lower gut microbiome diversity in GDM mothers compared to healthy controls during pregnancy, with differences varying by trimester and clinical confounders (Sun et al., 2023; Wang S. et al., 2024). In animal models, fecal microbiota transplantation (FMT) from GDM patients into pregnant mice induced characteristic changes in the gut microbiota of both the mice and their offspring, such as a significant reduction in *Akkermansia* abundance. These findings support the concept of intergenerational microbial transmission in experimental settings (Qin et al., 2022).

#### 2.4.2 Postpartum dynamics and the modulating effects of delivery and feeding modes

Maternal GDM is also associated with higher rates of cesarean delivery, which is a critical modifier of vertical microbial transfer. During vaginal birth, the infant is exposed to the maternal vaginal and fecal microbiota, acquiring pioneer bacteria such as *Bacteroides* and *Victivallis*. Cesarean delivery bypasses this natural inoculation, leading to the enrichment of environmental genera (e.g., *Veillonella*), reduced acquisition of maternal microbial strains, and increased potential for antibiotic resistance gene carriage (Chieu et al., 2024; Dualib, Fernandes et al., 2022). In GDM pregnancies, where maternal gut dysbiosis already exists, cesarean delivery further disrupts the already compromised vertical transmission pipeline, potentially amplifying the offspring’s exposure to a dysbiotic microbial community.

Breastfeeding practices are also often affected by GDM. Women with GDM experience delayed lactogenesis and shorter breastfeeding duration more frequently than normoglycemic women. Beyond these clinical challenges, GDM also alters the composition of breast milk itself. Li X. et al. (2023) demonstrated that alterations in human milk oligosaccharides (HMOs) in mothers with GDM impede the colonization of beneficial bacteria such as Bifidobacterium and disrupt the development of RORγt+ Treg cell-mediated immune tolerance in neonates. Furst et al. (2026) further reported that GDM-associated changes in HMO concentrations are associated with infant weight gain. Exclusive breastfeeding normally promotes the colonization of *Bacteroides* and *Staphylococcus*, contributing to microbiota stability and immune development (Dualib, Fernandes et al., 2022). Therefore, both the reduced duration of breastfeeding and the altered milk composition in the GDM group may attenuate these protective effects and allow the persistence of pathogenic or pro-inflammatory taxa transmitted from the mother.

These delivery and feeding characteristics are not merely parallel modifiers; they directly interact with GDM-associated vertical transmission. Notably, the similarity of gut microbiota between GDM mothers and their infants varies more widely than in healthy dyads and correlates negatively with maternal BMI. Furthermore, the influence of delivery mode and infant sex on microbial structure can even surpass that of GDM itself, underscoring that clinical management of GDM must account for these confounding routes (Chieu et al., 2024; Petitclerc et al., 2025) (Table 3).

**Table 3:** Alterations in gut microbiota-derived metabolites in GDM.

Studies	Area	Subjects	Altered metabolites	Research method
Gao et al. (2022)	China	GDM patients (*n* = 24)	Increased: isobutyric acid, isovaleric acid, valeric acid, caproic acid, GUDCA, THDCA + TUDCA, LCA-3S	GC–MS UPLC–MS
Tao et al. (2023)	China	GDM patients (*n* = 71)	Increased: Leucine, Isoleucine, Valine, Phenylalanine, Butyrate, TMAO, Glucose Citrate, 3-Hydroxybutyrate Decreased: Tyrosine, Isobutyrate	NMR spectroscopy
Qiu et al. (2025)	China	GDM patients (*n* = 269)	Increased: Triglycerides, HbA1c, C-peptide Decreased: Linoleic acid, Adiponectin	GC–MS
Li et al. (2025)	China	GDM patients (*n* = 40)	Increased: total n-6 PUFA, saturated fatty acids (SFA) Decreased: total PUFA, total n-3 PUFA, C18:3n-3	GC
Hu Y. et al. (2025)	China	Neonates (*n* = 15)	Increased: IL-6, CRP, LPS, PCT Decreased: acetate, butyrate, propionate, Isovaleric acid	16S rDNA GC–MS
Peterson et al. (2025)	Estonia	Children (*n* = 38)	Increased: anti-β-lactoglobulin IgA antibodies Decreased: IgA, IgG	Flow cytometry, Immunoblot
Han et al. (2024)	China	GDM patients (*n* = 49)	Increased: Iso-butyrate Decreased: butyrate/iso-butyrate, Butyrate	GC–MS
Wu et al. (2023)			Increased: Phenylacetate, Serine, Indoleacetate, Adrenate Decreased: Pyruvate, Pipecolate, Carnitine Glycodeoxycholate	GWAS data
Wang J. et al. (2024)	China	GDM patients (*n* = 321)	Increase: Choline Decrease: betaine, carnitine	HPLC–MS/MS
Yao et al. (2024)	China	GDM patients (*n* = 201)	Increased: Choline Decreased: L-carnitine	LC-MS
Zheng et al. (2025)	China	GDM patients (*n* = 70)	Increased: Leucine, Isoleucine, Valine, Phenylalanine, Tyrosine, p-Cresol, p-Cresol sulfate, Indoxyl sulfate, Benzoic acid, TMAO, Betaine etc. Decrease: Phenylacetylglycine, Phenylacetate, Lactate, Choline etc.	Untargeted metabolomics
Susarla et al. (2024)	America	GDM patients (*n* = 91)	Increased: carbocyclic acids, branched-chain amino acids clusters, 4-hydroxyphenylacetic acid, leucine, isoleucine, valine, oleic acid, linoleic acid, arachidonic acid	Untargeted metabolomics
			Decreased: Hydrogenated cinnamic acid	

The impact of GDM on maternal and infant microbiota is time-dependent and significantly modulated by delivery and feeding modes. In mothers, the overall gut microbiota composition of GDM mothers was significantly altered at 2 months postpartum (independent of BMI), characterized by increased abundances of *Dialister* and *Butyricicoccus* and a decreased abundance of *Oscillospiraceae* (Petitclerc et al., 2025). In infants, the effect of GDM exposure is not consistently stable. No significant microbial differences were observed at 1 month of age. However, by 6 months, infants exposed to GDM showed reduced microbiota diversity, enrichment of *Actinobacteria*, depletion of *Proteobacteria*, and these alterations were correlated with growth indices (Song et al., 2023). In contrast, a study by Chieu et al. indicated that the functional impact of GDM on the infant gut microbiome was moderate and transient. By 12 months of age, only a slight reduction in alpha diversity was observed, suggesting that early effects might be compensated for by later developmental processes (Chieu et al., 2024). These observations highlight that vertical transmission in GDM is a multifactorial process heavily shaped by clinical birth practices and postnatal care.

#### 2.4.3 Alterations in offspring gut microbiota and long-term health risks

Most studies indicate that exposure to GDM is associated with a series of alterations in the diversity, composition, and function of the offspring’s gut microbiota, which are associated with long-term health risks. Regarding microbial characteristics, multiple studies report significantly reduced alpha and beta diversity in the gut microbiota of neonates born to GDM mothers. At the phylum level, this is often characterized by decreased abundances of Actinobacteria and Firmicutes, alongside an increased abundance of Proteobacteria. At the genus level, beneficial bacteria (e.g., *Bifidobacterium*, *Blautia*, *Faecalibacterium*, *Roseburia*) show reduced abundances, while potential pathogens or pro-inflammatory bacteria (e.g., *Stenotrophomonas*, *Chryseobacterium*, *Pseudescherichia*, *Escherichia-Shigella*) exhibit increased abundances (Hu Y. et al., 2025; Mo et al., 2025; Zhang, Tan et al., 2025). Su et al. (2025) found that neonates of GDM mothers exhibited reduced alpha diversity and altered beta diversity in their meconium microbiota. They also showed decreased abundances of *Firmicutes* and *Eubacterium.* (including *Eubacterium hallii*), along with significant changes in metabolite levels such as fumarate and succinate. This suggests that maternal GDM may affect early microbial colonization and metabolic programming in offspring. This early microbial disturbance may pose long-term health risks. Research suggests that GDM, especially when combined with maternal overweight /excessive gestational weight gain, may increase the future risk of inflammatory diseases, metabolic disorders (e.g., childhood obesity), and neurodevelopmental disorders in offspring by altering neonatal microbial colonization (Athalye-Jape et al., 2025; Xiao et al., 2024; Zhu et al., 2022). A recent review further highlighted that GDM-induced gut dysbiosis may contribute to adverse fetal neurodevelopmental outcomes, underscoring the importance of microbiome-targeted precision medicine approaches (Biete and Vasudevan, 2024). For example, reduced abundances of *Burkholderia-Caballeronia-Paraburkholderia* and *Enterobacteriaceae* in the meconium of GDM-exposed neonates were associated with increased body mass index (BMI) at 12 months of age (Zhu et al., 2022). A higher abundance of the SCFA-producing genus *Fusicatenibacter* at 18 months was significantly associated with lower total behavioral problem scores (Nieto-Ruiz et al., 2023). Furthermore, GDM exposure may lead to delayed maturation of the offspring’s gut microbiota. This is evidenced by reductions in *Veillonella*, increases in *Bacteroides*, impaired vitamin B12 metabolism functions, and a significantly lower accuracy in microbiota age prediction (Valdez-Palomares et al., 2024).

Nevertheless, heterogeneity exists in the findings from this field. Some studies have observed different patterns. For instance, one study found no significant difference in neonatal gut microbiota alpha diversity between groups, but a significant separation in beta diversity. It reported an increased abundance of Bacteroidetes alongside decreased abundances of Actinobacteria and Proteobacteria, as well as an enrichment of opportunistic pathogens such as *Bacteroides* and *Escherichia-Shigella* (Zhang, Tan et al., 2025). Another study, based on 247 meconium samples from a Chinese population, revealed that the early gut microbiota composition was similar between neonates born to GDM mothers and those born to mothers with normal blood glucose (GLU). Differences were only observed in the abundance of specific taxa and in diversity indices (Liu et al., 2025). These discrepancies may arise from variations in study populations, research design, disease characteristics, and methodological approaches. Current observational evidence cannot fully exclude confounding factors. Future efforts should rely on large-scale, standardized birth cohort studies combined with animal model experiments to clarify the characteristics and mechanisms of GDM’s impact on offspring gut microbiota, as well as its causal relationship with long-term health outcomes. In order to better understand whether early gut microbiota dysbiosis caused by GDM has lasting effects or is only a temporary effect, future studies should track observations from infancy through childhood. Monitoring “microbiome age” as well as metabolic and neurodevelopmental outcomes will be particularly valuable. Such longitudinal data can help clarify whether early microbiota dysbiosis leads to persistent changes in metabolic programming or neurodevelopment, or whether its effects diminish as the child grows.

## 3 Metabolomic mechanisms and biomarkers of gut microbiota influencing GDM

### 3.1 Characteristic alterations in the metabolic profiles of GDM mothers and infants

GDM is not only associated with changes in maternal circulating metabolic profiles but may also affect offspring through alterations in breast milk composition and is directly reflected in the metabolic features of newborns. These changes collectively depict a multidimensional landscape of GDM-associated metabolic dysregulation. Regarding maternal circulating metabolites, GDM patients exhibit significant alterations in microbiota-related metabolites, though the direction of change varies by metabolite function. On one hand, levels of certain gut microbiota-derived metabolites are elevated in the serum of GDM pregnant women, including specific SCFAs (such as isobutyrate, isovalerate, valerate, and caproate) and bile acids like glycoursodeoxycholic acid and taurohyodeoxycholic acid (Gao et al., 2022). On the other hand, metabolites positively associated with the risk of type 2 diabetes and cardiovascular diseases, such as BCAAs and trimethylamine N-oxide (TMAO), are also enriched in the GDM group serum, with most differential metabolites closely linked to gut microbiota metabolism (Tao et al., 2023). Furthermore, elevated plasma levels of linoleic acid (LA) during pregnancy are significantly associated with a reduced risk of GDM. This effect may be modulated by the gut microbiota, as LA-related microbiota may mediate its association with C-peptide, and the abundance of *Bilophila* has been found to modify the strength of the LA-GDM relationship. Concurrently, increased LA is associated with elevated adiponectin and decreased triglycerides, HbA1c, and C-peptide, suggesting potential crosstalk between systemic and gut microbial metabolism (Qiu et al., 2025). Alterations in maternal metabolic state extend to breast milk composition, potentially contributing to offspring microbial dysbiosis. A prospective cohort study found that increased levels of C18:3n-3 and total n-3 polyunsaturated fatty acids (PUFAs) in breast milk were negatively correlated with changes in specific infant gut bacterial phyla (e.g., *Acidobacteriota*, *Gemmatimonadota*, *Myxococcota*). Compared to the normal group, the increase in these n-3 PUFAs over time was significantly lower in the breast milk of GDM mothers. This difference was associated with gut microbiota dysbiosis in GDM-exposed offspring, indicating that insufficient n-3 PUFAs (particularly C18:3n-3) in breast milk may contribute to GDM-associated offspring gut dysbiosis (Li et al., 2025; Peterson et al., 2025). Regarding offspring metabolic features, neonates exposed to GDM already show alterations in early metabolic programming. Compared to neonates of healthy pregnant women, those born to GDM mothers have significantly lower fecal levels of acetate, propionate, butyrate, and isovalerate, suggesting impaired microbial fermentation function (Hu Y. et al., 2025). This early metabolic environment may affect immune system development. A study of 88 children (aged 1–6 years) born to mothers at high risk for GDM found a significantly reduced antibody response (IgG MFI and number of IgA/IgG bands) to *Clostridium butyricum* strain T2F3. The offspring’s antibody response was influenced by maternal BMI, the child’s own allergic status, and HLA genotype. This suggests that maternal GDM may influence the offspring’s immune response to beneficial bacteria and supports the potential anti-allergic role of *Clostridium butyricum* (Peterson et al., 2025) (Table 4). Current research has limitations, including limited sample sizes, predominantly cross-sectional designs, a lack of dynamic tracking, and non-standardized methods, raising questions about the generalizability of metabolite features across populations. Furthermore, the causal relationship between metabolites and microbiota, the molecular mechanisms of breast milk’s influence on offspring, the long-term health risks associated with offspring abnormalities, and their quantitative links with clinical indicators remain to be clarified. Heterogeneity across metabolomic studies is driven primarily by differences in detection platforms (GC–MS, NMR, LC–MS), sample types (serum vs. feces vs. milk), and the extent of control for dietary intake and antibiotic exposure. These factors limit direct comparison of metabolite alterations across studies.

**Table 4:** Correlations between gut microbiota/metabolites and clinical indicators in GDM.

Studies	Significantly altered gut microbiota and metabolites	Associated clinical indicators/functions	Correlation/diagnostic value
Xiao et al. (2024)	*Coriobacteriaceae, Collinsella*	Gestational weight	Negative correlation
Liang et al. (2022)	*Paraprevotella, Roseburia, Faecalibacterium, Ruminococcaceae_UCG-002*	GLU	Negative correlation
	Ruminococcaceae_UCG-002	Hemoglobin A1c	Negative correlation
	Bacteroides	GLU	Positive correlation
Dualib, Fernandes et al. (2022)	Christensenellaceae, Intestinobacter	1 h GLU	Positive correlation
	Enterococcus	2 h GLU	Negative correlation
Vavreckova et al. (2022)	*Escherichia/Shigella*	Plasma lipid	Positive correlation
	Subdoligranulum	Plasma lipid	Negative correlation
Sun et al. (2023)	F/B *ratio, A. putredinis*	FPG	Negative correlation
	*Eubacterium ramulus*	FPG	Positive correlation
Wang J. et al. (2024)	*Akkermansia muciniphila, Coprococcus eutactus, Enterorhabdus caecimuri, Dialister* sp. *CAG 357*	2 h OGTT	Negative correlation
Gao et al. (2022)	Isobutyric acid, Isovaleric acid, Valeric acid, Caproic acid, GUDCA, THDCA + TUDCA, LCA-3S	Early diagnosis value	AUC = 0.890
Tao et al. (2023)	Leucine, Isoleucine, Valine, Phenylalanine, Butyrate	Diagnosis value	AUC = 0.988
	Phenylacetate, Galactonic acid, Isobutyrate, Phenylacetylglycine	1 h OGTT	Negative correlation
	Hydroxyphenyllactic acid, 5-hydroxyindoleacetic acid, citrate	1 h OGTT	Positive correlation
	Phenylacetate, galactonic acid, isobutyrate, phenylacetylglycine, lactate, tyrosine	2 h OGTT	Negative correlation
	Taurine, 2-hydroxybutyrate, butyrate	2 h OGTT	Positive correlation
Qiu et al. (2025)	*Haemophilus*, *Erysipelotrichacea*e	LA	Positive correlation
	*Bilophila, Klebsiella*	LA	Negative correlation
	D-altronate dehydratase	Leptin, FBG	Positive correlation
	D-altronate dehydratase	HDL cholesterol	Negative correlation
Li et al. (2025)	Acidobacteriota, Gemmatimonadota, Myxococcota, *Sphingomonas*, *Allorhizobium, Neorhizobium, Pararhizobium etc.*	total n-3 PUFA, C18:3n-3	Negative correlation
Hu Y. et al. (2025)	*Bifidobacterium, Blautia, Anaerostipes, Faecalibacterium etc.*	IL-6, CRP, LPS, PCT	Negative correlation
	*Stenotrophomonas, Chryseobacterium, Pseudescherichia*	IL-6, CRP, LPS, PCT	Positive correlation
Peterson et al. (2025)	*Clostridium butyricum*	Respiratory system	Positive correlation
	*Clostridium butyricum*	Atopic dermatitis	Negative correlation
	Maternal BMI	children’s IgG	Positive correlation
Han et al. (2024)	T1: Butyrate/iso-butyrate	Predictive value	AUC = 0.81
	Butyrate	Predictive value	AUC = 0.73
	T2: iso-butyrate	Predictive value	AUC = 0.96
Wu et al. (2023)	*Collinsella, Coprobacter*, *Olsenella*	GDM risk	Positive correlation
	*Oscillibacter, Methanobrevibacter*	Predictive value	Positive correlation
	Serine, indoleacetate, adrenate, phenylacetate	GDM risk	Positive correlation
	Pyruvate, Pipecolate, Carnitine Glycodeoxycholate	Protective	Positive correlation
Wu Y. et al. (2025)	Choline	GDM risk	Positive correlation
	Betaine, carnitine	GDM risk	Negative correlation
Yao et al. (2024)	Choline	GDM risk	Positive correlation
	L-carnitine	GDM risk	Negative correlation
Zheng et al. (2025)	*Alistipes*	Glucolipid	Negative correlation
	*Enterobacter, Anaerotruncus, Delftia*	Post-load glucose	Positive correlation
	2-piperidinone	GDM risk	Positive correlation
	Oleoyllinoleoyl-glycerol (18:1/18:2)	GDM risk	Negative correlation
Susarla et al. (2024)	Alpha-aminoadipic acid, arachidic acid, glucose, carbocyclic acids, unsaturated fatty acid cluster	GDM risk	Positive correlation
	Hydrocinnamic acid	GDM risk	Negative correlation
	Conventional risk factors (age, BMI, history of diabetes etc.), 11 gut microbial metabolites (trans-4-hydroxy-l-proline, 4-imidazoleacrylic acid, butyric acid, 2,6-diaminopimelic acid etc.)	Predictive value	AUC = 0.884

### 3.2 Potential of metabolites as biomarkers for GDM

The gut microbiota and its host-derived metabolites are not only closely associated with the risk of GDM, but also show significant value in early prediction and diagnosis of the disease. Related research mainly focuses on the association between specific metabolites and risk, as well as the construction of prediction models based on metabolites. Regarding the association between metabolites and GDM risk, SCFAs produced by gut microbial metabolism show specific correlations. Butyrate levels are negatively correlated with GDM risk, while isobutyrate levels (particularly in the second trimester) are positively correlated. Furthermore, the butyrate/isobutyrate ratio demonstrates superior predictive performance compared to either single metabolite (Han et al., 2024). Additionally, phenylacetic acid (via phenylalanine metabolism), serine, and indoleacetate are positively associated with GDM risk, whereas pyruvate acts as a protective factor (Wu et al., 2023). Two separate studies consistently indicate that high choline levels are associated with an increased risk of GDM. One nested case–control study based on a Shanghai birth cohort further found that elevated betaine and carnitine levels were associated with a reduced GDM risk, with betaine’s protective effect being more pronounced in multiparous women (Wu Y. et al., 2025). Another study confirmed that high L-carnitine levels were linked to a lower GDM risk (Yao et al., 2024). However, neither study established a genetic causal relationship between these metabolites and GDM. Untargeted metabolomics revealed that women with recurrent GDM exhibited significant downregulation of caffeine metabolites (e.g., caffeine, theobromine, paraxanthine) in early pregnancy and dysregulation of lipid metabolites (e.g., 15-Deoxy-Δ^12^,^14^-prostaglandin J₂) in mid-pregnancy. These alterations were negatively correlated with postprandial glucose levels (Zheng et al., 2025). A multi-ethnic prospective study also found that clusters of carboxylic acids, BCAAs in early pregnancy, and unsaturated fatty acids in mid-pregnancy were positively associated with GDM risk (Susarla et al., 2024). In terms of diagnostic and predictive value, the metabolite model demonstrates high efficiency. Tao et al. (2023) constructed a random forest classifier based on 21 core metabolites (e.g., BCAAs, TMAO), which achieved an AUC of 0.988 for diagnosing GDM. Gao et al. (2022) reported that valerate (AUC = 0.831), a combination of THDCA and TUDCA (AUC = 0.814), and a panel of metabolite markers (AUC = 0.890) all showed good diagnostic value. Susarla et al. (2024) developed a model integrating metabolites with traditional risk factors, achieving a predictive AUC of 0.884–0.987, which outperformed models using single-type indicators (Tables 2, 4). Current research predominantly focuses on correlations between metabolites and GDM, lacking validation of genetic causality. Furthermore, the detection standards for metabolite biomarkers and their generalizability across different ethnicities and geographic regions remain unclear, limiting their clinical translation and application. Metabolite-based models face comparable translational challenges. External validation in independent cohorts is scarce, reproducibility across populations and detection platforms (e.g., GC–MS, NMR, LC–MS) has not been systematically evaluated, and the predictive value of metabolite signatures in early pregnancy has yet to be prospectively confirmed.

### 3.3 Molecular mechanisms of key metabolites in influencing GDM

Specific gut microbiota metabolites can participate in the onset and development of GDM through multiple mechanisms, including regulating host signaling pathways, interfering with placental function, and affecting fetal development. Firstly, certain metabolites can directly modulate key host signaling pathways, influencing insulin sensitivity and inflammatory status. Gut microbial metabolites such as luteolin and naringenin chalcone can regulate the AGE-RAGE signaling pathway by targeting proteins like AKT1, thereby affecting inflammatory responses. This provides potential targets for metabolite-based interventions (Sabarathinam et al., 2025). Secondly, an unhealthy dietary pattern, such as a high-fat diet, can induce gut dysbiosis, triggering a cascade of metabolic disturbances that have been implicated in GDM. Studies show that dysbiosis induced by a high-fat diet (e.g., enrichment of the pro-inflammatory bacterium *Alistipes* and reduction of beneficial bacteria like *Akkermansiaceae*) is accompanied by disordered bile acid metabolism. These alterations collectively activate the hepatic Fxr-Shp-Fgf15 signaling pathway, exacerbating systemic insulin resistance and contributing to GDM progression (Yao L. et al., 2025). Furthermore, gut dysbiosis reduces the production of SCFAs, such as propionate and butyrate. This reduction inhibits the intestinal HDAC3-H3K27ac-PPAR-*γ* signaling axis and activates the fatty acid transporter CD36, facilitating the release of lipids into the systemic circulation and aggravating peripheral insulin resistance (Chen et al., 2024). Among these, SCFAs, as a class of core metabolites, exert adverse effects on both the mother and fetus when their levels are decreased through various pathways. At the maternal-fetal interface, reduced circulating levels of SCFAs (especially butyrate) in GDM mothers inhibit placental GPR41/43 receptors and activate histone deacetylases. This may promote localized placental inflammation and disrupt its normal metabolic function (Wang et al., 2022). Moreover, evidence suggests the impact of this metabolic dysregulation may extend to fetal organ development. Emerging evidence indicates that decreased maternal SCFA levels attenuate the activation of the GPR43 receptor in fetal kidney cells, subsequently impairing cell proliferation and migration. This mechanism may be associated with an increased risk of congenital kidney and urinary tract anomalies (Wang H. et al., 2025).

## 4 Intervention strategies targeting the gut microbiota in GDM

### 4.1 Lifestyle and nutritional interventions

Lifestyle and nutritional modifications form the cornerstone of GDM management, with their benefits being partly mediated through the modulation of gut microbiota. The following sections will discuss the effects and limitations of dietary adjustments, physical activity, and integrated interventions.

Regarding dietary interventions, various approaches can exert effects by modulating the gut microbiota. After short-term dietary intervention or medical nutrition therapy, pregnant women with GDM show increased abundances of beneficial bacteria such as *Ganoderma*, *Roseburia*, and *Bifidobacterium*, alongside decreased abundances of harmful bacteria associated with hyperglycemia like *Hanseniaspora*, *Zygosaccharomyces*, and *Desulfovibrio*. These changes were accompanied by improvements in glucose metabolism (Chen et al., 2022; Wu et al., 2022). A diet high in complex carbohydrates increases the abundance of *Bifidobacterium* in GDM women, which is negatively correlated with fasting blood glucose and triglycerides. This dietary pattern also improves infant early-life microbial diversity and reduces opportunistic pathogens like *Enterococcus* (Sugino et al., 2022). A high intake of vegetables and fruits is significantly associated with a lower risk of GDM. The protective effect of this dietary pattern is mediated by specific bacteria, with *Lachnospiraceae*, *Blautia*, and *Ruminococcus* accounting for 45.81, 44.33, and 31.53% of this mediation effect, respectively (Shan et al., 2024). The role of specific dietary components is also closely linked to the microbiota. A Chinese birth cohort study combined with Mendelian randomization analysis confirmed a causal negative association between circulating LA levels during pregnancy and GDM risk. This protective effect may involve the gut microbiota and appears stronger in individuals with lower *Bilophila* abundance, suggesting that LA dietary interventions should consider individual microbial profiles (Qiu et al., 2025). An Iranian study indicated that the intake of carbohydrates, monounsaturated fatty acids, and PUFAs promotes the proliferation of *Bacteroidetes*, while daily caloric and cholesterol intake is positively correlated with gut microbiota dysbiosis in GDM (Sohrabi et al., 2025). Furthermore, the efficacy of dietary interventions can be validated through microbiota transplantation. FMT from GDM women post-intervention into germ-free mice improved the mice’s glucose tolerance (Frishman et al., 2024). However, in cases of unsuccessful dietary intervention, neonates of these GDM mothers showed a reduced abundance of *Clostridiales* in their first stool and a significantly higher F/B ratio in both meconium and first stool, suggesting potential vertical transmission of maternal dysbiosis (Huang et al., 2021).

Lifestyle and nutritional supplementation interventions also demonstrate potential for modulating the gut microbiota. Engaging in 200 min of exercise per week can increase gut microbiota alpha diversity in GDM patients, elevating beneficial bacteria like *Faecalibacterium* and reducing the proportion of *Actinobacteria* (Xu et al., 2025). Conversely, high meat/iron intake, low physical activity, and poor sleep quality in women with GDM have been associated with a decrease in genera such as *Victivallis* and *Lactiplantibacillus*, affecting SCFA synthesis and insulin sensitivity (Kunasegaran et al., 2024). A randomized controlled trial (RCT) demonstrated that early-pregnancy supplementation with probiotics and dietary fiber-fermented milk significantly reduced the incidence of GDM in overweight/obese pregnant women by enriching beneficial bacteria like *Bifidobacterium* and *Acidovorax* while suppressing genera such as *Megasphaera*. However, an intensified diet and lifestyle intervention alone, although reducing gestational weight gain, did not significantly lower GDM risk (Zhang L. et al., 2025). In GDM patients with good glycemic control, lifestyle intervention led to a significant increase in the abundances of *Bifidobacterium adolescentis* and *Bifidobacterium longum*, which were negatively correlated with blood glucose levels and positively correlated with plasma SCFA levels (Cui et al., 2024). Notably, while combined dietary and exercise interventions can effectively improve glycemic control and pregnancy outcomes in GDM women and are associated with specific alterations in the gut microbiome and serum metabolome, they do not significantly alter the overall structure of the gut microbiota (Cheng et al., 2025). Furthermore, the gut microbial network structure in GDM patients does not shift toward a healthier state following dietary intervention, and individual network deviations are correlated with abnormal blood glucose regulation (Liu Y. et al., 2023). Although lifestyle and nutritional interventions can improve GDM by modulating the gut microbiota, their clinical translation still faces multiple challenges. These challenges include significant individual variability in effectiveness influenced by factors such as baseline gut microbiota and genetics, a lack of long-term follow-up evidence on the future health of both mothers and infants, the difficulty in restoring the overall gut microbiota to a state comparable to healthy pregnancy, and the absence of standardized, quantifiable microbiota monitoring indicators for real-time assessment and strategy adjustment. Collectively, these factors limit precise clinical application.

Collectively, dietary and lifestyle modifications are supported by multiple RCTs and remain the first-line clinical strategy for GDM management. However, their effects on the gut microbiota are often modest and subject to substantial inter-individual variability, highlighting the need for adjunctive microbiota-targeted approaches.

### 4.2 Probiotics, prebiotics, and synbiotics

Probiotics, prebiotics, and their combination as synbiotics can all participate in GDM intervention by modulating the gut microbiota and related metabolic pathways. However, the efficacy of different formulations varies across populations. Among microbiota-targeted interventions, probiotics and synbiotics have the strongest clinical evidence base in GDM, with multiple double-blind RCTs and meta-analyses demonstrating improvements in glycemic control.

#### 4.2.1 Probiotics

Probiotics demonstrate microbiota-modulating and metabolic-improving effects in both mother-infant GDM cohorts and animal models. A double-blind RCT on infants born to GDM mothers showed that a 4-month supplementation with three *Bifidobacterium* strains increased the abundance of beneficial bacteria like *Bifidobacterium* and reduced pathogenic bacteria such as *Gammaproteobacteria* in infant guts. It also enhanced microbial diversity and fecal SCFA content, potentially exerting positive effects on growth and neurodevelopment (Athalye-Jape et al., 2025). In an RCT involving women with prior GDM at 4–8 weeks postpartum, a 12-week multi-strain probiotic intervention increased the abundance of *Bifidobacterium adolescentis* and reduced Gram-negative bacteria like *Bacteroides fragilis*. This optimized the microbial structure and improved glycemic control, lipid profiles, and inflammatory status (Hasain et al., 2022). In animal experiments, supplementation with high-dose *Lactobacillus rhamnosus* LGG and *Bifidobacterium animalis* subsp. *lactis* Bb12 regulated metabolic pathways to ameliorate abnormalities in GDM rat models (Zheng et al., 2022). Oral administration of *Bifidobacterium* increased the proportion of immunoregulatory cells and decreased pro-inflammatory cytokines in GDM model animals (Liang et al., 2023). Furthermore, the probiotic *Pediococcus acidilactici* CECT 9879 and its heat-inactivated postbiotic form lowered blood glucose and alleviated insulin resistance. This was achieved by modulating the gut microbiota (e.g., increasing *Pediococcus acidilactici* and *Lactobacillus plantarum*, decreasing *Streptococcus infantarius*), improving hepatic insulin signaling (upregulating *insulin receptor*, *peroxisome proliferator-activated receptor alpha*), and regulating lipid metabolism (promoting *β*-oxidation, inhibiting lipogenesis) (Yavorov-Dayliev et al., 2025).

#### 4.2.2 Prebiotics

Prebiotics can individually modulate the gut microbiota and related pathways to influence GDM, although discrepancies exist between human and animal study results. A RCT on galacto-oligosaccharides (GOS) found that prenatal GOS supplementation did not improve maternal glucose/lipid metabolism or reduce GDM risk, but it did increase the abundance of *Paraprevotella* (Wan, An et al., 2023). In contrast, animal studies showed that GOS could activate the PPARs/PI3K/Akt pathway, regulate SCFA and bile acid metabolism, and improve blood lipid profiles and multi-organ damage in GDM model animals (Wan, Zhu et al., 2023). The prebiotic xylooligosaccharides (XOS), acting synergistically with *Akkermansia muciniphila*, enhanced intestinal barrier function and suppressed the NKG2D/NKG2DL signaling pathway, significantly improving insulin resistance in GDM mice (Wang J. et al., 2024). Supplementation with a mixture of XOS and inulin may modulate host metabolism by promoting the proliferation of *Bifidobacterium* (Wang Y. et al., 2025). Alginate oligosaccharides increased the abundance of *Akkermansia*, alleviated oxidative stress, and improved hyperglycemia and pancreatic damage in GDM mice (Zhang P. et al., 2025).

#### 4.2.3 Synbiotics

Studies on the intervention effects of synbiotics in women with GDM have shown some variation across individual trials. However, systematic reviews integrating multiple RCTs provide a more comprehensive insight into the potential roles of probiotics and synbiotics. A double-blind RCT showed that GDM women at 24–28 weeks of gestation, who received a 6-week daily supplementation of a synbiotic containing *L. acidophilus*, *L. plantarum*, *L. fermentum*, *L. gasseri*, and fructo-oligosaccharides, exhibited a significant reduction in the logTG/HDL-C ratio and an increase in HDL-C levels. However, no significant changes were observed in high-sensitivity C-reactive protein (hs-CRP), malondialdehyde (MDA), or other atherosclerosis-related markers. This suggests that this synbiotic may reduce future cardiovascular disease risk in GDM women by modulating lipid metabolism, although these effects require validation in studies with larger sample sizes and longer intervention periods (Nabhani et al., 2022). Another RCT with a similar design found that a 6-week intervention with a synbiotic supplement containing specific *Lactobacillus* strains and fructo-oligosaccharides did not significantly affect fasting blood glucose or insulin resistance/sensitivity indices in GDM women. However, it increased HDL-C and total antioxidant capacity within the group and significantly reduced blood pressure (Nabhani et al., 2018). A systematic analysis of 11 RCTs indicated that probiotic or synbiotic supplements (with synbiotics containing specific strains and prebiotics like inulin showing more pronounced effects)could significantly improve glucose metabolism markers [e.g., fasting blood glucose, fasting serum insulin, and homeostatic model assessment of insulin resistance (HOMA-IR)] and the lipid marker total cholesterol in women with GDM. These benefits are likely mediated by modulating the gut microbiota (e.g., increasing *Bifidobacterium* abundance, lowering the F/B ratio) and related metabolites like SCFAs (Mu et al., 2023). A meta-analysis encompassing 8 RCTs also noted that in GDM patients, probiotic use reduced fasting blood glucose, fasting insulin, and HOMA-IR levels, while synbiotic use reduced HOMA-IR. Both hold promise for assisting glycemic control, although more high-quality studies are needed to evaluate their safety (Çetinkaya Özdemir et al., 2022). The clinical application of probiotics, prebiotics, and synbiotics still faces multiple obstacles, mainly including the lack of standardized evidence-based strains, dosages, and treatment protocols, which leads to high heterogeneity in studies. Most existing evidence comes from short-term trials, and the long-term safety for mothers and infants as well as the long-term health benefits for offspring remain unclear. The effectiveness of interventions is significantly influenced by the host’s existing gut microbiota, resulting in considerable individual differences. In the future, predictive models based on individual microbiota characteristics need to be developed to achieve precise interventions.

### 4.3 FMT: preclinical evidence and translational barriers

Animal studies have preliminarily confirmed the potential of FMT in GDM intervention. Transplanting fecal microbiota from healthy donor mice into GDM model mice significantly lowered the recipient’s blood glucose levels. It also restored serum levels of SCFAs, including butyrate, propionate, and acetate. These preclinical findings suggest that FMT may improve glycemic status by restoring SCFA metabolism (Wang H. et al., 2025). However, current research remains confined to animal models, lacking evidence from clinical human trials. The safety, suitable target populations, and long-term effects of FMT are still undefined. More prospective clinical studies are needed to evaluate its practical application value in GDM management.

**Figure 2:**
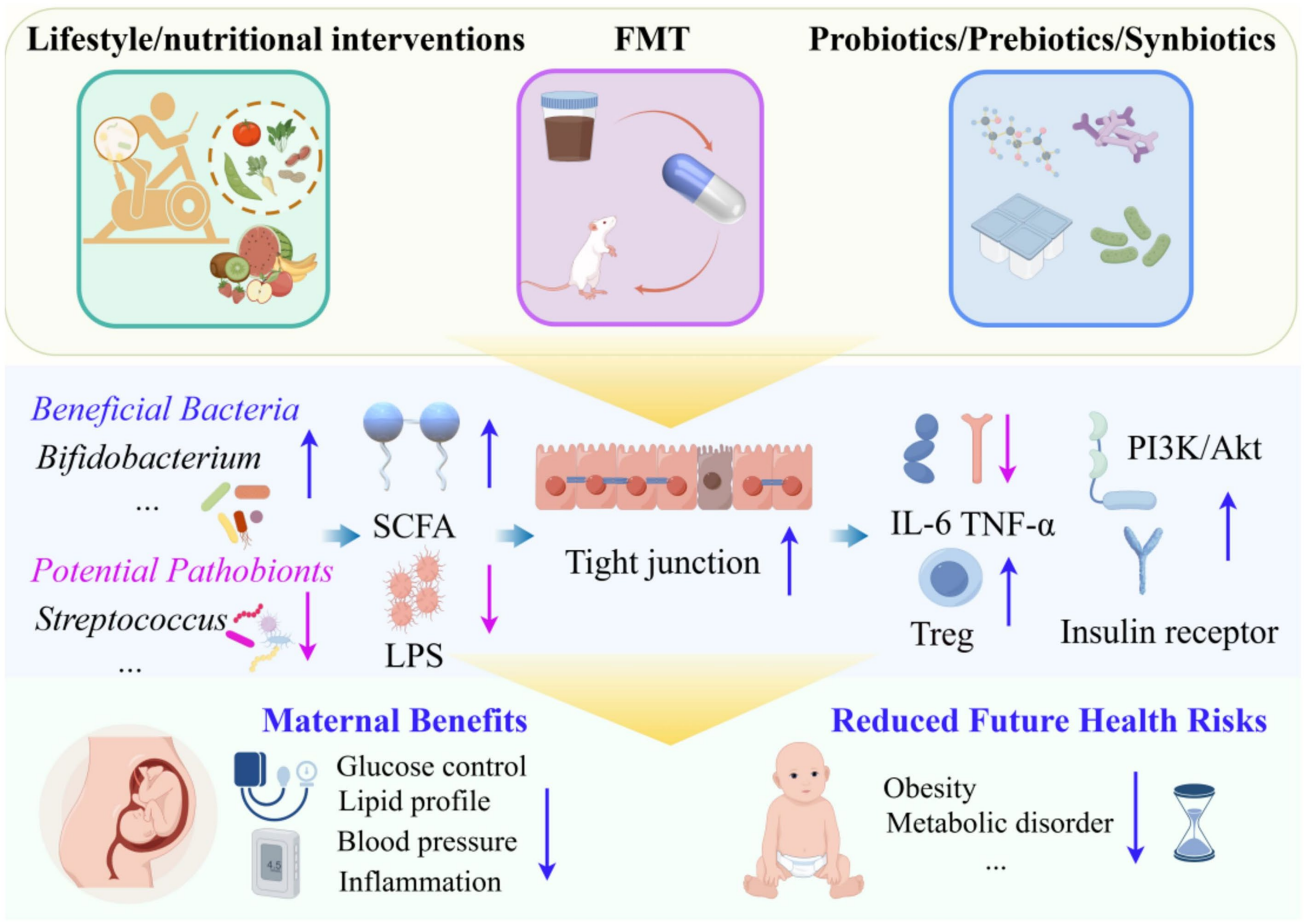
The therapeutic strategies of GDM based on gut microbiota. (By Figdraw).

Several major barriers preclude the current application of FMT in GDM management. First, the safety profile of FMT during pregnancy has not been established; potential risks include maternal infection, immune activation, and unintended transfer of pathogenic microorganisms to the fetus. Second, no standardized donor screening protocols exist for pregnant recipients, and the criteria for defining a “healthy” pregnancy microbiota remain undefined. Third, the stability and engraftment of transplanted microbiota in the context of pregnancy-related hormonal and immunological changes have not been studied. Fourth, the ethical and regulatory frameworks for administering FMT during pregnancy are undeveloped. Given these unresolved concerns, FMT should be regarded at present as a mechanistic research tool rather than a therapeutic intervention for GDM. Its clinical translation pathway will require extensive preclinical safety data and carefully designed phase I trials in non-pregnant populations before any consideration of use during pregnancy (Figure 2).

## 5 Sources of heterogeneity across studies

The inconsistent findings across GDM gut microbiota and metabolomic studies likely reflect several overlapping sources of heterogeneity. Population diversity across study sites (e.g., China, Colombia, Brazil, Estonia, Singapore, United States) represents a primary contributor, as distinct dietary patterns, genetic backgrounds, and environmental exposures shape the gut microbiota independently of GDM status. Gestational age at sampling is a second major source: the gut microbiota and circulating metabolome shift substantially across trimesters, and studies sampling at different gestational windows are not directly comparable.

Technical differences further complicate cross-study comparisons. For microbiota studies, shotgun metagenomics provides species-level and functional resolution, whereas 16S rRNA gene sequencing is generally limited to genus-level taxonomic assignment. For metabolomic studies, detection platforms (e.g., GC–MS, NMR, LC–MS) and analytical strategies (targeted vs. untargeted) vary considerably in sensitivity, coverage, and compound identification confidence. The extent of control for confounding factors also differs markedly: pre-pregnancy BMI, antibiotic exposure, dietary intake, and, in infant studies, delivery mode and feeding modality are not consistently accounted for across studies. Finally, sample sizes range from fewer than 20 to more than 300 participants, with smaller studies carrying higher risks of spurious findings. These methodological differences collectively limit the interpretability and comparability of results across the current literature. A detailed summary of study-level heterogeneity factors is provided in Tables 1, 3, 4.

## 6 Conclusion and perspectives

The gut microbiota serves as a crucial interface connecting host metabolism, immunity, and endocrine networks, playing a pivotal role in the onset and progression of GDM and its intergenerational impact on maternal and infant health. Current evidence indicates that women with GDM exhibit characteristic gut microbiota dysbiosis and alterations in related microbial metabolites. These changes may contribute to the disease process by interfering with insulin sensitivity, compromising barrier function, and activating inflammatory responses. Furthermore, they may also influence offspring microbial colonization and long-term health risks through vertical transmission. Diagnostic models based on microbial features and microbiota-targeted intervention strategies (e.g., dietary modifications, probiotics) show promising potential. However, this field still faces significant challenges, including high heterogeneity across studies, unclear causal mechanisms, and difficulties in clinical translation. As recently proposed in a microbiome-informed preventive framework, rethinking GDM from a microbial perspective could fundamentally shift current prevention strategies (Turjeman et al., 2026).

Translating these research findings into clinical practice requires a structured roadmap. The first set of challenges is technical: external validation of microbiome- and metabolome-based biomarkers in multi-center, independent cohorts remains limited, and reproducibility across populations and analytical platforms (16S rRNA vs. shotgun metagenomics; GC–MS, NMR, LC–MS) has not been systematically demonstrated. Integration with conventional clinical markers such as fasting glucose, HbA1c, OGTT results, and pre-pregnancy BMI may improve predictive performance and clinical utility, but optimal combination strategies are yet to be defined. The feasibility of first-trimester risk prediction, the most clinically relevant window, also requires further prospective investigation, as many current models are based on samples collected later in pregnancy. Beyond these technical issues, several practical considerations warrant attention. Regulatory pathways for microbiome-based diagnostic tools need clarification, including U.S. Food and Drug Administration and Conformité Européenne marking requirements and clinical validation standards. Cost-effectiveness analyses are essential to justify clinical implementation, particularly for low-risk populations. Addressing these interconnected challenges will be critical to move microbiome-based tools from discovery to routine antenatal care.

Future research should integrate large-scale prospective cohorts with multi-omics technologies, artificial intelligence, and machine learning models. Combined with Mendelian randomization analyses and animal experiments, these approaches can help systematically elucidate the mechanisms by which core microbiota influence GDM. On the one hand, constructing dynamic networks of interactions between microbiota and host can help analyze associations among microbial functions, inflammatory factors, and genetic background, and verify robust biomarkers. On the other hand, based on individual baseline microbiota characteristics, genetic background, and lifestyle, personalized intervention plans and precise prediction tools covering pre-pregnancy, pregnancy, and postpartum periods can be developed. Beyond metagenomics, metatranscriptomics and metaproteomics offer valuable additional layers. Metagenomics shows which microbes and genes are present; metatranscriptomics reveals active gene expression; and metaproteomics identifies functional proteins produced. Together, these approaches move from associations toward causal mechanisms. Ultimately, this would enable a translational leap from basic research to precise prevention and clinical management of GDM, improving short-term maternal–infant outcomes and blocking intergenerational transmission of metabolic diseases. This represents a core strategic direction with both clinical and public health value.
